# Changes in Musculoskeletal System and Metabolism in Osteoporotic Rats Treated With Urocortin

**DOI:** 10.3389/fendo.2019.00400

**Published:** 2019-06-24

**Authors:** Dominik Saul, Laura Katharina Geisberg, Torben Gehle, Daniel Bernd Hoffmann, Mohammad Tezval, Stephan Sehmisch, Marina Komrakova

**Affiliations:** ^1^Department of Trauma, Orthopedics and Reconstructive Surgery, Georg-August-University of Göttingen, Göttingen, Germany; ^2^Klinik für Unfallchirurgie, Sporttraumatologie und Handchirurgie, Klinikum Vest, Recklinghausen, Germany

**Keywords:** osteoporosis, urocortin, muscle, bone, metabolism

## Abstract

**Objective:** In aging population, postmenopausal osteoporosis and decline of musculoskeletal function, referred to as “frailty syndrome” lead to loss of bone and muscle, causing falls, and fall-related injuries. To limit the impact of this portentous duo, simultaneous treatment of both is needed. Urocortin (UCN) has been reported to improve osteoporotic bone properties while its effect on muscle has not been addressed yet.

**Design and Methods:** We aimed to investigate the effect of urocortin *in vivo* on skeletal muscle structure in osteopenic rats. Sixty Sprague-Dawley rats were divided into five groups: four were ovariectomized (OVX) and one underwent sham operation (SHAM). One ovariectomized group was left untreated (OVX), while one was treated with urocortin s.c. in 3 μg/kg body weight (bw) (OVX+UCN low), one with 30 μg/kg (OVX+UCN high), while one group was treated with estradiol orally (OVX+E: 0.2 mg/kg bw), each for 35 days. *Mm. gastrocnemius, longissimus*, and *soleus* were isolated and capillary density as well as diameters of type I and II fibers were measured. In addition, we examined the effect of UCN on tibia using biomechanical, micro-CT and ashing analysis and investigated the blood serum.

**Results:** We demonstrated a positive effect of UCN on *M. soleus*, in which fiber diameter was positively influenced. The biomechanical and structural parameters of bone were not changed in UCN treated rats. The higher cholesterol, glucose and triglyceride levels in the “UCN high” group raise concern about this treatment.

**Conclusions:** Our results portray urocortin as a substance that can be assessed for future therapeutic treatments of estrogen deficiency.

**New and Noteworthy:** Urocortin has a positive effect on *M. soleus* (diameter). Urocortin raises serum cholesterol and triglyceride levels. Bone tissue was not affected by UCN.

## Introduction

Due to the high prevalence of postmenopausal osteoporosis (1, 2), a strong muscular function is essential for the protection of the incorporated bone and physical coordination to prevent falls. In the postmenopausal women, estrogen deficit does not only cause osteoporosis, but was also related to muscular dysfunction (3, 4). As “muscle bone interactions” are focused on more and more and the incidence of osteoporosis and sarcopenia is closely linked, we aimed to assess whether in a rat model for postmenopausal osteoporosis skeletal muscle could be addressed (5–7). In recent decades and with a distinct diagnostic definition, multiple therapeutic options and breakthroughs have been developed for osteoporosis (8, 9), whereas muscle-related research has not proceeded as far (10, 11). Post-menopausal estrogen replacement therapy (ERT) was often recommended for prevention and treatment of osteoporosis (12) as well as for maintaining muscle strength and mass (13, 14). However, ERT is associated with potential side effects on reproductive tissues (12). Current potential therapeutic options against muscle loss include human recombinant bone morphogenetic protein (BMP)-2/7 and anti-myostatin antibodies (15–17), though certain of the latter (activin decoy receptor IIB: ActRIIB-Fc) seem to elevate bone mass (18–20). The effect of vitamin D supplementation remains inconclusive, although beneficial effects on muscle strength and prevention of falls in sarcopenic post-menopausal women have been reported (21). Recently, creatine supplementation has been proposed to positively affect sarcopenic state in aging postmenopausal women, while effect on bone remains inconclusive (22).

Hence, innovative therapeutic strategies against muscle loss need to address both, bone and muscular systems.

Urocortin I (UCN), a peptide consisting of 40 amino acids (23), and its fellow members of the corticotropin-releasing factor peptide family positively affect the cardiovascular system *in vitro* (24–28) and *in vivo* (29, 30) and modulate stress responses as well as ingestive behavior (31). Urocortin I exerts its effects via the corticotropin-releasing factor 1 and 2 receptor (CRF1R and CRF2R) and has a 40-fold higher affinity than corticotropin-releasing factor (CRF) itself at CRF2Rs (32, 33). Since UCN has received more and more attention as a cardio-protective agent (34, 35) and seems to be effective at cutting excessive food consumption (36–38), further effects of these promising peptides need to be elucidated.

The positive UCN effects on osteoporotic femur (39) and spine (40) have been recently demonstrated by our group, but analysis of skeletal muscle and metabolic status has not been performed yet. Promising results, however, have been published by Hinkle et al. (41), where urocortin II treatment, like the CRF2R-agonists urocortin I and sauvagine, decreased the casting-induced loss of muscle mass *in vivo*. However, no further characterization of muscle fibers or capillarization has been conducted so far. Since immobilization-induced and infarction-induced muscle loss was positively influenced by UCN (42), we wanted to test whether this effect could also be observed in skeletal muscle of an osteoporotic rat.

To mimic the clinical situation, in which muscle changes often accompany bone loss (43–45), we chose the ovariectomized rat as an established model of postmenopausal osteoporosis (46), in which UCN could simultaneously affect impaired bone tissue along with the muscle (47). In the present study, the tibia was analyzed to confirm previously reported positive effects (39, 40) in bone. *M. gastrocnemius* (both slow and fast fiber types) and *M. soleus* (mainly slow fiber types) as lower leg agonists were chosen as surrogates for different fiber types, while *M. longissimus* (with mainly fast fiber types) represents a holding muscle. We sought to investigate the changes in muscle fibers and capillarization as parameters of nutrient supply (48, 49) of several skeletal muscles in osteoporotic rats after subcutaneous administration of urocortin in different doses. Since fiber diameter alterations alone do not sufficiently represent muscle structure and function, quotients between capillaries and fibers were calculated and metabolic muscle enzymes were measured (50). Moreover, the metabolic status of the rats was evaluated.

## Materials and Methods

### Animals and Treatment

In this study, 60 female Sprague-Dawley rats (Winkelmann Company, Borchen, Germany) were kept at 22°C with 55% relative humidity in Makrolon IV®-Cages. One acclimatization week was included prior to the beginning of the experiment, which took place in conformance with the ethical standards of animal care. The experimental design was approved by the local institutional animal care and use committee (district authorities of Oldenburg, Germany, registration numbers: 33.9-42502-04-10/0246).

At an age of 13 weeks, the rats underwent bilateral ovariectomy (OVX) or sham surgery (SHAM) as described elsewhere (51). Operations were carried out under ketamine (90 mg/kg body weight (bw), Hostaket®, Hoechst, Bad Soden, Germany) and xylazine anesthesia (7.5 mg/kg bw, Rompun®, Bayer, Leverkusen, Germany) applied 0.1 ml/100 g bw intraperitoneally. After shaving and disinfection, skin was incised below the ribs on both sides, and the abdomen was opened. Adnexa were prepped and the tubae uterinae tied up. Ovaries were removed with a scalpel before wound closure.

Based on previous studies, around 8 weeks after OVX, the rats were expected to develop osteoporosis with concomitant hormone withdrawal (46, 52, 53). From week eight, we started the UCN treatment. Urocortin (urocortin [human] trifluoroacetate salt, 96% purity [HPLC], Bachem Bubendorf, Switzerland), dissolved in 300 μl 0.9% NaCl, was injected subcutaneously at the same time each day for 35 days at either 1.05 μg/rat (3 μg/kg bw, OVX+UCN low group) or 10.5 μg/rat (30 μg/kg bw, OVX+UCN high group). For comparability, the groups SHAM, OVX and OVX+Estradiol were injected with 300 μl 0.9% NaCl subcutaneously at the same times.

The OVX+Estradiol group was supplied via food (10 mg/kg food) with 0.2 mg/kg bw estradiol-17-benzoate (Sigma-Aldrich Chemie GmbH, Taufkirchen, Germany) per day for 35 days. All rats received a soy-free diet (Ssniff special diet GmbH, Soest, Germany) during the experiment. Food intake and body weight were recorded once weekly and are already published (39, 40). In the end, rats were CO_2_-anesthetized and decapitated.

### Tissue Isolation and Processing

Rats were sacrificed after 35-day treatments. Uterus, heart, liver, kidneys, spleen and visceral adipose tissue were extracted and weighed. *Mm. gastrocnemius* and *soleus* were weighed and transversely cut in half across the whole muscle. A piece of *M. longissimus* (1 × 3 cm) was extracted. After that, the muscles were snap-frozen in liquid nitrogen and stored at −80°C. Afterwards, 12 μm frozen sections were cut orthogonally with a cryotome (Leica 2800E Frigocut Microtome Cryostat, Leica Biosystems, Nussloch, Germany).

### Staining

**Figure 1:**
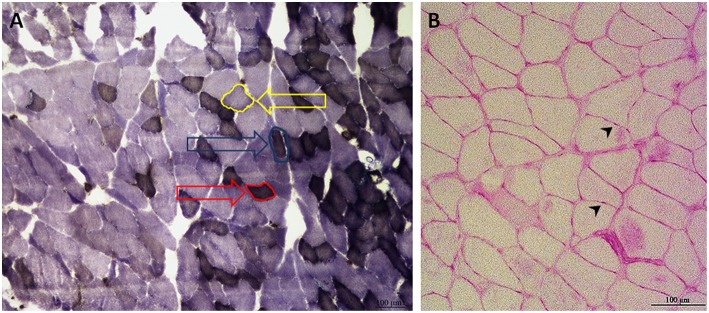
Exemplary ATPase **(A)** and PAS **(B)** staining of *M. gastrocnemius* **(A)** and *M. soleus* **(B)**. The ATPase staining of *M. gastrocnemius* differentiates three types of muscle fibers: type I fibers are red-framed with a red arrow. Type IIa fibers are edged in blue and marked with a blue arrow. Type IIb fibers are yellow-rimmed with a yellow arrow **(A)**. Type IIa and Type I were combined for the analysis. The PAS staining of *M. soleus*, with capillaries marked by black arrowheads **(B)** (100 × magnification).

To distinguish type I from type II muscle fibers, adenosine triphosphatase (ATPase) staining was carried out as described elsewhere (54). Three different image areas were chosen within the histological section of each muscle. Therein, 30 type I and 30 type IIb fibers were outlined, and the mean diameter was measured with the help of Lucia G image analysis system (Version 4.82, Laboratory Imaging, Prague, Czech Republic). Because slow oxidative fibers (type I) were rarely represented and cannot always be clearly distinguished from fast-twitch oxidative glycolytic fibers (IIa), they were analyzed combined in *M. gastrocnemius* and M-longissimus (type I) (Figure 1A) (52). In *M. soleus*, most of the fiber types are type I (55). Therefore, no further differentiation was conducted, and solely type I fibers were analyzed.

For the relationship of fiber to capillaries, periodic acid-Schiff (PAS) staining was performed, following the method of Andersen (56, 57). Two areas of 500 × 500 μm were chosen within the muscle section, wherein fibers and capillaries were counted and their ratio calculated (Figure 1B).

The selection of image areas as well as their analyses have been performed by one individual who was blinded with respect to the treatment groups.

### Analysis of Muscle Enzymes

Muscle specimens were first assimilated in Chappell-Perry medium (0.1 M KCl, 0.05 M Tris, 0.01 M MgCl_2_ × 6H_2_O, 1 mM EGTA, pH 7.5). Afterwards, they were assessed with a spectrophotometer (Spectronic Genesys 2PC, Pittsford, USA). The assay was performed within 2 h following the tissue homogenization. The lactate dehydrogenase (LDH) activity was indirectly measured by recording the reduction in absorption at 340 nm, which demonstrates the oxidation of NADH to NAD^+^ (58).

Citrate synthase (CS) activity was measured at 412 nm with Triton X-100 (0.1%) for the liberation of all CS proteins according to Faloona and Srere (59), whereas the complex I activity was assessed at 340 nm before the complex I inhibitor rotenone was added to the incubation solution for measuring the NADH reduction, as reported by Hatefi and Stiggall (60). Protein content was measured with a multilabel reader (Perkin Elmer Precisely Victor X4, Turku, Finland) and software version 4.0 (Perkin Elmer Life and Analytical Science). The activity of enzymes was calculated relative to the protein content. Detailed protocols have been previously described by our group (61–63).

### Biomechanical Analysis

Tibia biomechanics was assessed using a Zwick device (145 660 Z020/TND; Ulm, Germany) as previously described (64). Four screws inside the metallic cylinders helped to fix the tibia on the metal plate as explained in Komrakova et al. (65). The bending test was stopped before plastic deformation ended; measurements were performed with a 5 mm/min motion rate and stopped at a curve-decline more than 2 N. Recording was conducted with testXpert software (Zwick GmbH & Co. KG, Ulm, Germany). The highest force that the tibia could resist was the maximum load (Fmax). The turnaround point from elastic to plastic deformation was referred to as the yield load (yL), while elasticity of the tibia (stiffness (S)) was measured as the slope of the linear rise of the curve during elastic deformation. All these values were quantified using Microsoft Excel (MS Office 2016) (66, 67).

### Micro-CT

The metaphyseal tibia was scanned with a micro-CT device (eXplore Locus SPScanner, GE Healthcare, Ontario, Canada) with 72 kVp, 90 μA, and 1,600 ms exposure time and 360° rotation, resulting in 0.0029 mm pixel size. Five hydroxyapatite blocks with defined mineral densities were scanned to transform gray scale values into bone mineral density (mg/cm3). 3D OsteoAnalyze, which was developed for our laboratory, was used to determine bone parameters according to American Society for Bone and Mineral Research (ASBMR) criteria (40, 68–70). Total, trabecular and cortical BMD (g/cm^2^) as well as bone volume fraction (BV/TV) were assessed at the metaphysis. Therefore, two measurements next to the growth plate were performed (3 and 5 mm), and the area in between was cut out. The volume and density of cortical, trabecular and total bone were measured in this field. After turning 90°, the cross-section was used to measure the distal cortical bone area (Ct.Ar), endosteal area (E. Ar), and total area (T. Ar.) of the bone. Thereafter, the cortical bone was cut off, and trabecular structure was analyzed. Trabecular thickness (Tb.Th), number of trabecular nodes (N.Nd), trabecular number (Tb.N), mean trabecular junctions at one node (Tb.N/Nd), and trabecular separation (Tb.Sp) were measured (69, 71).

### Ashing

The tibia was ashed at 750°C for 1 h. Afterwards, calcium content was quantified with an atomic absorption spectrometer (4100; PerkinElmer, Baesweiler, Germany). Orthophosphate content was assessed by the colorimetric method (Spectral Photometer DM4; Zeiss, Jena, Germany) (67, 72). The inorganic weight was measured after ashing as a percentage of the wet weight before ashing (39).

### Serum Analyses

Serum analyses were conducted at the Department of Clinical Chemistry, University of Goettingen according to the manufacturers' instructions (Abbott, Wiesbaden, Germany).

After collecting 5 ml of whole blood from the rats in the end of the experiment, it was centrifuged at 3,000 × g for 10 min. Serum was stored at −20°C. Creatine kinase (CK), Alanine transaminase (ALT), triglycerides, uric acid, Aspartate aminotransferase (AST/GOT), Cholesterol, glucose, and HDL were measured with the ARCHITECT c System and AEROSET System (Abbott, Wiesbaden, Germany).

### Statistics

Statistical analysis was performed with GraphPad Prism (5.04, GraphPad Software, Inc. San Diego, CA). One-way ANOVA (F test, α = 0.05) was conducted to reveal the impact of the treatments on variables. For variables that showed an overall statistically significant difference in group means (*p* < 0.05, F test), the Tukey-Kramer *post hoc* test (α = 0.05) was performed to estimate the differences between individual means (Supplemental Table 1). Mean values and standard error of the mean (SEM) are shown in all figures.

## Results

### Rat Characteristics

In the beginning of the study, body weight of rats did not differ among the groups, whereas at the end of the treatments, the mean weight of the SHAM (324.8 ± 23.5 g) and OVX+Estradiol (324.3 ± 16.5 g) groups was significantly lower compared to the OVX (396.8 ± 18.9 g), OVX+UCN high (388.3 ± 43.7 g) and OVX+UCN low (375.2 ± 16.6 g) groups [*p* < 0.05; (39, 40)]. Body weight in the OVX+UCN high and OVX+UCN low groups did not differ from that in the OVX group. As expected, the uterus was significantly lighter in all ovariectomized animals, i.e., OVX, OVX+UCN high, and OVX+UCN low groups, compared to those in the SHAM and OVX+Estradiol groups, as shown previously by our group [*p* < 0.05; (39, 40)]. Though the weight of the uterus increased after estradiol treatment, it was still lower than in the SHAM group.

**Figure 2:**
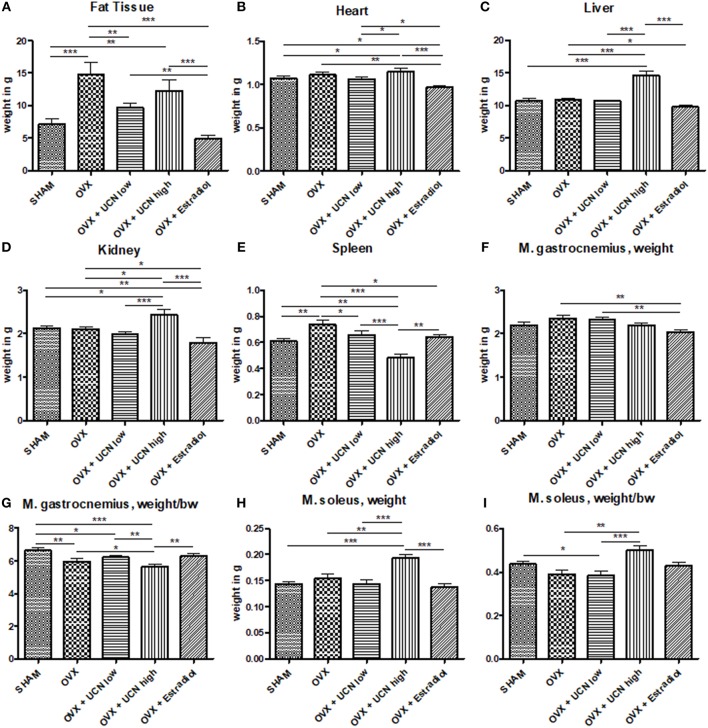
Weight of organs and muscle tissue at the end of the experiment. Organ tissues were measured, exemplarily fat tissue **(A)**, heart weight **(B)**, liver weight **(C)**, kidney weight **(D)**, and spleen weight **(E)**. Additionally, total weight of *M. gastrocnemius* **(F)** and *M. soleus* **(H)** as well as their ratio to body weight **(G,I)** were assessed. Data are shown as mean ± SEM. *p < 0.05; **p < 0.01; ***p < 0.001. SHAM *n* = 11; OVX *n* = 10; OVX+UCN low *n* = 12; OVX+UCN high 12; OVX+Estradiol *n* = 12.

Visceral fat mass was most abundant in the OVX and OVX+UCN high groups (Figure 2A). Regarding heart weight, OVX+Estradiol group showed the lowest weight compared to all other groups (Figure 2B). In OVX+UCN high group, heart weight was higher than that in SHAM and OVX+UCN low groups. OVX+UCN high led to the highest results in the liver weight, which could also be seen in the kidney weight (Figures 2C,D). OVX+UCN high had the lowest spleen weight compared to all other groups (Figure 2E). In *M. soleus*, OVX+UCN high caused significantly higher values compared to all other groups (Figure 2H). OVX and OVX+UCN low showed higher total *M. gastrocnemius* weight compared to OVX+Estradiol (Figure 2F). Regarding the ratio of *M. gastrocnemius* weight to body weight, OVX+UCN high caused lowest ratio, while SHAM led to a significantly higher ratio compared to all (except OVX+Estradiol) treatments (Figure 2G). In weight/bw-ratio of *M. soleus*, OVX+UCN high caused significantly higher ratio compared to OVX+UCN low and OVX (Figure 2I). Taken together, there was an effect of UCN treatment on inner organs and fat.

Superficial observational analysis of rat activity did not reveal differences regarding daily levels of exercise. Movements among groups were comparable and treatment with UCN did not cause any gain or decrease of action.

### Fiber Diameter in Muscles

**Figure 3:**
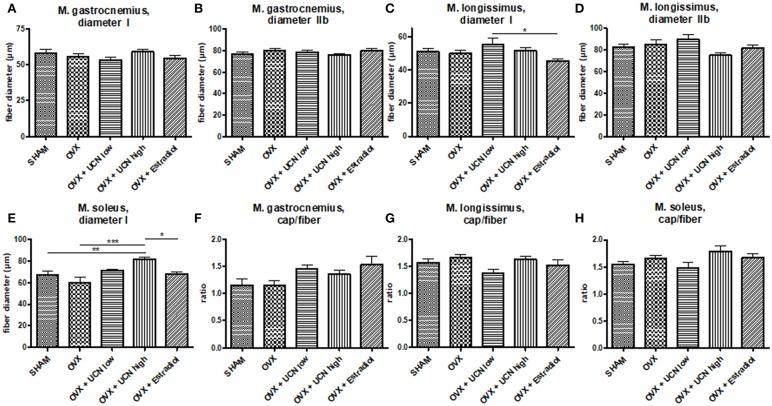
Muscle fiber diameter **(A–E)** was measured microscopically in *M. gastrocnemius* **(A,B)**, *M. longissimus* **(C,D)** and *M. soleus* **(E)** in fiber type I **(A,C,E)** and IIb **(B,D)**. The ratio of capillaries per fiber was counted **(F–H)**. Data are shown as mean ± SEM. *p < 0.05; **p < 0.01; ***p < 0.001. SHAM *n* = 9; OVX *n* = 6; OVX+UCN low *n* = 9; OVX+UCN high 10; OVX+Estradiol *n* = 8.

Next, to evaluate the effect of urocortin treatment on muscle, we decided to measure muscle fiber parameters in *Mm. gastrocnemius, longissimus*, and *soleus*. In *M. gastrocnemius*, fiber diameter was homogeneous between the all groups. With a mean diameter of 53.3–59.0 μm, no differences in type I fiber diameter were detected (Figure 3A). In fiber type IIb, mean diameter ranged from 75.9 to 79.6 μm, also without any significant differences (Figure 3B).

In *M. longissimus*, the diameter of type I fibers was significantly smaller in OVX+Estradiol (45.3 μm) compared to OVX+UCN low (55.3 μm) (Figure 3C). Type IIb fibers showed no difference between OVX+UCN low (89.60 μm) and OVX+UCN high (75.16 μm) (Figure 3D).

In *M. soleus*, the mean diameter of fibers was significantly higher in OVX+UCN high (81.6 μm) compared to SHAM (67.0 μm), OVX (59.9 μm), and OVX+Estradiol (68.2 μm) (Figure 3E). Effect of UCN on fiber diameter was most evident in *M. soleus*, whereas in *Mm. gastrocnemius* and *longissimus*, the UCN effect was not significant.

### Ratio of Capillaries to Muscle Fibers

To explain the differences in muscle characteristics, we investigated the capillary supply. No statistically significant differences were detected between groups in the ratio of capillaries to *M. gastrocnemius* (Figure 3F) or *M. longissimus* fibers (Figure 3G). In *M. soleus*, no differences could be detected too (Figure 3H).

### Muscle Enzyme Activity

**Figure 4:**
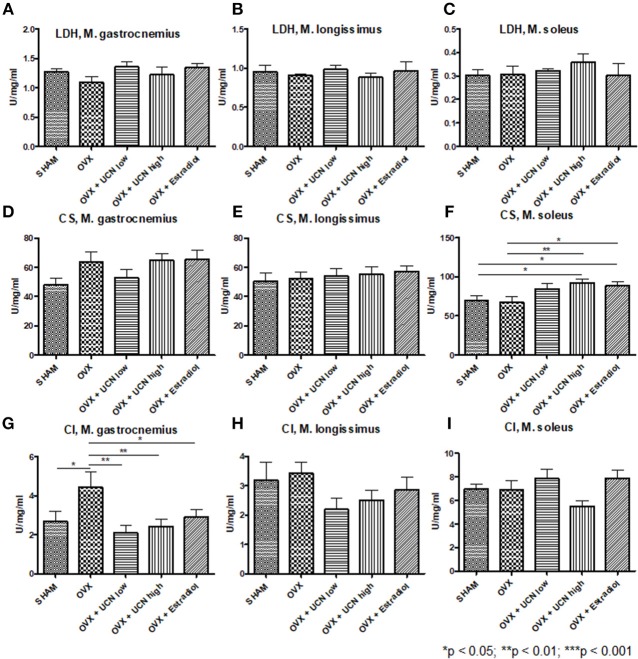
Analysis of muscle enzyme activity. Activity of lactate dehydrogenase (LDH, **A–C**), citrate synthase (CS, **D–F**), and complex I (CI, **G–I**) was measured using spectrophotometry in *M. gastrocnemius* **(A,D,G)**, *M. longissimus* **(B,E,H)**, and *M. soleus* **(C,F,I)**. Data are shown as mean ± SEM. *p < 0.05; **p < 0.01; ***p < 0.001. SHAM *n* = 8; OVX *n* = 8; OVX+UCN low *n* = 8; OVX+UCN high 8; OVX+Estradiol *n* = 8.

For metabolic insights, we measured enzyme activity in the abovementioned muscles. Lactate dehydrogenase (LDH) activity in *M. gastrocnemius, M. longissimus*, and *M. soleus* was not different between the groups (Figures 4A–C).

Regarding citrate synthase (CS) activity, no differences could be obtained in *M. gastrocnemius* (Figure 4D) as in *M. longissimus* (Figure 4E). In *M. soleus*, the OVX+UCN high and OVX+Estradiol groups showed significantly higher CS activity compared to the SHAM and OVX groups (Figure 4F).

Complex I (CI) activity showed the highest levels in OVX compared to all other groups in *M. gastrocnemius* (Figure 4G), whereas in *Mm. longissimus* and *soleus*, no differences could be detected (Figures 4H,I).

Enzyme activity in terms of citrate synthase was significantly enhanced by UCN high treatment, whereas UCN therapy did not lead to differences in LDH or CI activity.

### Biomechanical Assessment of Tibia and Ashing

After detection of metabolic muscle alterations, we proceeded to assess bone parameters to learn whether the former lead to changes in the subjacent bone. While stiffness was not altered, yield load and maximum load were significantly diminished in the OVX+UCN low group compared to SHAM and OVX+Estradiol. In the OVX group, yield load and maximum load were lower than in the SHAM group (Table 1).

**Table 1:** Biomechanical, ashing, and micro-CT analyses of the tibia in SHAM-operated rats and OVX rats either untreated or treated with urocortin at different concentrations (UCN low, UCN high) or estradiol.

**Sample size**	**SHAM**		**OVX**		**OVX+UCN low**		**OVX+UCN high**		**OVX+Estradiol**	
	**Mean**	**SEM**	**Mean**	**SEM**	**Mean**	**SEM**	**Mean**	**SEM**	**Mean**	**SEM**
**BIOMECHANICS**										
Stiffness [N/mm]	170	31	147	30	144	29	164	31	157	29
Yield load [N]	141	20	116^a^	22	105^a, c^	12	123	19	129	13
Maximum load [N]	142	20	116^a^	22	106^a, c^	13	124	19	130	13
**ASHING**										
Ca^2+^ [mmol/m]	0.46	0.01	0.45	0.01	0.44^a^	0.00	0.44^a^	0.00	0.45	0.00
PO_4_3− [mmol/m]	0.29	0.00	0.28	0.00	0.29	0.00	0.28	0.00	0.3	0.01
Ca^2+^/P${\text{O}}_{4}^{3-}$	1.18	0.02	1.59	0.03	1.53	0.01	1.54	0.00	1.51	0.04
Inorganic mass (%)	49.86	0.32	47.95^a^	0.35	47.45^a^	0.15	48.76^a, b^	0.25	48.73^a, b^	0.25
**MICRO-CT**										
Total BMD [mg/cm3]	604	11.39	477.8^a^	8.65	472.2^a^	9.03	482.0^a^	15.69	496.5^a^	11.19
Bone volume (BV, mm^3^)	9.73	0.52	3.75^a^	0.46	3.3^a^	0.44	4.34^a^	0.42	3.14^a^	0.33
Total volume (TV, mm^3^)	12.47	0.73	11.40	0.49	10.67	0.47	11.72	0.4	10.85	0.33
BV/TV [%]	78.38	1.17	32.8^a^	3.8	30.03^a^	3.31	37.31^a^	3.83	28.89^a^	3.05
Tr.+Ct. BMD [mg/cm3]	757.8	7.26	792.6	6.94	764.1	6.52	780.4	8.1	797.5^a^	12.57
Tr. BMD [mg/cm3]	613.4	8.53	567.7^a^	10.25	543.6^a^	7.22	574.5	13.62	582.8	10.6
Ct. BMD [mg/cm3]	952.8	7.19	915.6^a^	8.14	909.9^a^	5.757	936.1	7.56	942.8^b^	9.21
Tb.N. [n]	821	51.18	435.5^a^	51.5	394.0^a^	49.68	545.3^a^	37.1	391.1^a^	41.1
N.Nd. [n]	1,063	69.03	524^a^	68.19	470.7^a^	63.02	663.1^a^	48.76	465.8^a^	53.79
Tb.N/Nd (n)	2.53	0.01	2.32^a^	0.03	2.29^a^	0.03	2.34^a^	0.03	2.29^a^	0.04
Tb.Sp. [mm]	0.19	0.00	0.20	0.01	0.21	0.00	0.20	0.00	0.21	0.01
Tb.Th. [mm]	0.07	0.01	0.03^a^	0.00	0.03^a^	0.00	0.03^a^	0.00	0.03^a^	0.00
Ct.Ar. [mm^2^]	4.50	0.11	4.65	0.14	4.55	0.1	4.7	0.12	4.43	0.10
E.Ar. [mm^2^]	6.55	0.16	6.67	0.27	6.74	0.22	7.03	0.28	6.45	0.15
T.Ar. [mm^2^]	2.05	0.08	2.02	0.14	2.19	0.15	2.33	2.69	2.02	0.09

SHAM n = 12; OVX n = 10; OVX+UCN low n = 12; OVX+UCN high 12; OVX+Estradiol n = 12.

a p < 0.05 vs. SHAM.

b p < 0.05 vs. OVX+UCN low.

c p < 0.05 vs. OVX+Estradiol.

*BMD, bone mineral density; BV/TV, bone volume fraction; BW, body weight; Ct., Cortical; Ct.Ar, cortical bone area; E.Ar., endosteal area; N.Nd, trabecular nodes; NON-OVX, non-ovariectomy; OVX, ovariectomy; SEM, standard error of the mean; T.Ar., Trabecular area; Tb.N, trabecular number; Tb.N/Nd, mean trabecular junctions at one node; Tb.Sp., trabecular separation; Tb.Th, trabecular thickness; Tr., Trabecular; UCN, urocortin-I*.

In the ashing analysis, the calcium content was significantly lower in OVX+UCN low group and OVX+UCN high than in SHAM (Table 1), corresponding to the inorganic mass of the tibia, for which the OVX+UCN low group showed lower values compared to those of SHAM, OVX+UCN high and OVX+Estradiol (Table 1). The inorganic mass was significantly lower in all OVX groups than in the SHAM group. The phosphate content did not change significantly between the groups (Table 1).

UCN treatment did not cause biomechanical changes or mineral differences in bone of OVX rats.

### Micro-CT

As a next step we investigated the microstructure of tibiae. Notably, micro-CT analysis showed that all OVX groups, compared to SHAM, had reduced total bone mineral density (Table 1). Lower trabecular BMD appeared in the OVX+UCN low and OVX groups compared to SHAM (Table 1). Cortical BMD revealed that OVX+UCN low-values were significantly lower compared to SHAM and OVX+Estradiol (Table 1). Bone volume fraction was reduced in all OVX groups, similar to total BMD (Table 1). Regarding the analysis of trabecular structure, the trabecular thickness, nodes, number, and trabecular junctions at single node were significantly decreased in all OVX groups (Table 1). Cortical bone area, endosteal and total bone area were not altered (Table 1). Micro-CT measurements confirmed osteoporotic phenotype after OVX, while UCN treatment did not alter trabecular or cortical parameters of OVX rats.

### Serum Analyses

**Figure 5:**
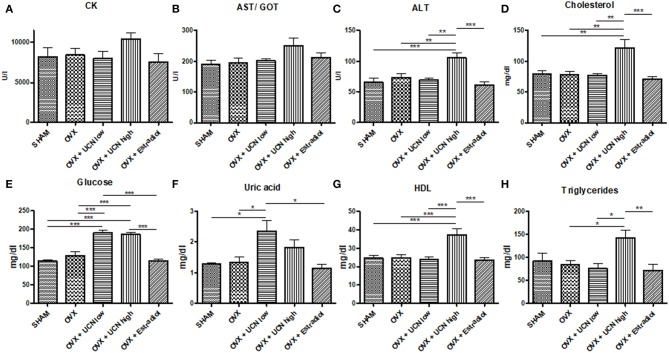
Serum values of creatine kinase (CK, **A**), aspartate transaminase/glutamic oxaloacetic transaminase (AST/GOT, **B**), alanine transaminase (ALT, **C**), cholesterol **(D)**, glucose **(E)**, uric acid **(F)**, High-density lipoprotein (HDL, **G**), and triglycerides **(H)** were measured at the end of experiment. Data are shown as mean ± SEM. *p < 0.05; **p < 0.01; ***p < 0.001. SHAM *n* = 5; OVX *n* = 7; OVX+UCN low *n* = 7; OVX+UCN high 7; OVX+Estradiol *n* = 7.

Finally, to assess the potential systemic impact of urocortin, investigation of serum parameters followed. Neither CK nor AST/GOT was significantly different among the groups (Figures 5A,B), though in the OVX+UCN high group the values were higher than in the other groups. Alanine transaminase was significantly increased in the OVX+UCN high group compared to all other groups (Figure 5C), as was serum cholesterol (Figure 5D). Glucose was significantly higher in both UCN-treated groups compared to SHAM, OVX and OVX+Estradiol (Figures 5E,F). UCN low showed the highest uric acid values, significantly higher than in SHAM, OVX, and OVX+Estradiol (Figure 5F). HDL-cholesterol was significantly higher in the OVX+UCN high group than all other groups (Figure 5G), and a similar result was seen in triglycerides (Figure 5H). Serum analyses demonstrated profound alterations, especially in ALT, cholesterol, glucose, and triglyceride levels, by UCN treatment.

## Discussion

To detect the impact of urocortin on musculoskeletal metabolism, we used an established osteoporotic rat model (65, 66). Five groups of rats were generated, two of which received different concentrations of urocortin (referred to as “UCN low” and “UCN high”), one was treated with Estradiol (OVX-Estradiol), one was just ovariectomized (OVX) and one was not (SHAM). The treatment regimens were chosen according to preliminary tests after literature review (41, 73).

Successful ovariectomy was demonstrated by the significantly raised body weight and reduced uterus weight in all OVX groups, while estrogen treatment lead to similar body weight compared to SHAM rats and an increase in uterus weight; UCN treatments did not alter body weight or uterus weight (40).

Muscle analysis was started with the largest muscle of the lower leg, *M. gastrocnemius*, which functions as a dynamic muscle because it contains more fast-twitch type IIb fibers in rats than in humans. In the slow-twitch type I fibers and type IIb fibers as well, no differences in diameter after treatments with urocortin were detected. The *M. soleus*, as a holding muscle, mainly consists of slow and constantly contracting type I fibers (46). Here, type I fibers were significantly thicker after higher urocortin treatment compared to the OVX group. These observations raise the question of cause and effect, which is not clear at this point.

Hinkle et al. (41) reported that urocortin could prevent loss of skeletal muscle mass. In their rat model, they analyzed *M. tibialis* anterior, *M. gastrocnemius*, and *M. soleus*. They used glucocorticoid, leg casting or nerve damage to induce muscle loss, while in our model, ovariectomy was performed. In both studies, urocortin was administered s.c. at a dose of 30 μg/kg (bw), while in our study, 3 μg/kg (bw, low) and 30 μg/kg (bw, high) were applied. In the study of Hinkle et al. (41), 10, 30, 100, and 300 μg/kg (bw) were used. The effect of urocortin on muscle mass in the study by Hinkle et al. (41) was more pronounced in the control group than in denervated or casted mice. The effect was stronger in *M. tibialis* anterior at a range of doses (30–300 μg/kg [bw]) than in *M. gastrocnemius*, in which the UCN did not inhibit muscle loss at any dose studied. In the glucocorticoid-induced muscle atrophy model, UCN inhibited muscle loss at all doses tested (41). In *M. extensor* digitorum longus and *M. soleus*, UCN treatment (300 μg/kg [bw]) inhibited casting-induced muscle mass loss, myocyte cross-sectional area loss and force loss (41). In our OVX model, we also observed enhanced muscle weight and muscle fiber size in *M. soleus* at the high dose and *M. longissimus* at the low dose. Therefore, we were able to demonstrate that urocortin has a positive effect on muscle mass even already at lower doses applied in our study.

In *M. longissimus*, which consists of approximately 20% type I fibers (46), we observed a significant decrease in type I fiber diameter in OVX+Estradiol group compared to the OVX+UCN low, but not between any UCN treatment and OVX. These results suggest a superior effect of the higher urocortin concentration on *M. soleus* compared to *M. longissimus*. The UCN high treatment exerted its greatest effects compared to SHAM and OVX in *M. soleus*, a possible hint that type I fibers benefit more from urocortin treatment than type IIb fibers do. Capillary density did not differ significantly between the groups among all muscle studied.

The analysis of creatine kinase and aspartate aminotransferase in serum, which can serve as an unspecific marker of muscle damage (74, 75), did not reveal significant differences between the groups, though the higher concentration of urocortin led to higher levels compared to all other groups. The impact of this substance on muscle damage may be questioned since a common marker of muscle loss, LDH, was not affected by UCN treatment in any muscle studied. Citrate synthase activity was higher in the OVX+UCN high group than OVX, which supports a favorable effect of UCN on muscle tissue, as is known for estradiol (76). However, the decreased activity of complex I in *M. gastrocnemius* after both UCN doses reflects the reduced mitochondrial activity and impaired possibility for an oxidative stress response (77) and should be evaluated in further studies.

The moderate effect of the urocortin treatment in this study can at least partially be attributed to the low dose administered and different animal model applied. An anabolic effect after urocortin I treatment on triceps surae muscles in dystrophic mdx (5Cv) mice has been seen at a dose of 300 μg/kg (bw) after 2 weeks (compared to our 35 days), which was 10-fold higher than the highest dose that we injected (78). Hinkle et al. (41) reported significant inhibition of muscle loss after 9-day UCN treatments in different atrophied muscle mouse models, which, however responded to the treatment differently. Whereas, glucocorticoid-induced muscle atrophy was inhibited at doses from 10 to 300 μg/kg (bw), the casting model was affected by doses higher than 100 μg/kg (bw). Furthermore, various muscles responded to the UCN treatment differently (41).

The adverse effects of urocortin on the cardiovascular system, with raised values of cholesterol, HDL and triglycerides, cause solicitude. Particularly, the impact of our highest UCN concentration on cholesterol has not been described in the literature. However, the cardio-protective effect of UCN is well-known (79–82). Our findings could nonetheless point out to some harming long-term effects of UCN. The results reached statistical significance in alanine transaminase (ALT), for which the high urocortin treatment group showed the highest values, indicating liver damage through this specific treatment. In a rat model with intracerebroventricular administration of UCN (0.01–1 nmol/day), these effects have also been reported after a 13-day treatment (83). The effects on glucose metabolism as the raised uric acid in the lowest-urocortin-concentration have also been described elsewhere (84). Heart, liver and kidney weight were also raised, which could indicate another side effect of urocortin treatment. The reduced spleen weight after the higher urocortin dose reveals once more the broad effect of urocortin, which we do not completely understand to this point.

Since, after entering blood circulation, the potential site of action for UCN are osteoblasts and osteoclasts as well as myocytes and immune cells (85, 86), we performed an analysis of bone parameters.

Regarding bone parameters, biomechanical evaluation revealed reduced yield load and maximum load in the UCN low group compared to SHAM and OVX+Estradiol group, similar to that in the OVX group. In the UCN high group, the biomechanical properties of bone were at the level of SHAM and Estradiol groups. Similar results were demonstrated in the inorganic mass in the ashing analysis, confirming the results of our group on rat femora and vertebrae, where UCN showed overall positive effects on bone parameters (39, 40). One possible mechanism could be the inhibition of osteoclast differentiation and function described by Combs et al. (87).

These results are, however, contradicted by the calcium levels measured in our study, where both UCN groups had the lowest levels. More precise evaluation of tibia with micro-CT did not confirm the harmful effects of low-dose urocortin treatment on trabecular or cortical bone, but it also did not confirm the positive effects that have been reported for high-dose urocortin treatment in spine and femur (39, 40). A possible explanation could be that effects of UCN are site specific, which has never been described before, but bone loss and microarchitecture changes in OVX rat models were described to be site specific (88). An explanation for the differential muscle changes could be that due to the less bone response underneath, the lower leg muscles did not show the same effect as *M. quadriceps femoris* would above the femur, which was not examined in our study. Contradictory, *M. longissimus* did not show significant effects despite spine did.

We conclude that the effects of urocortin I seem to be fiber-type specific and that type I fibers benefit more from the treatment. Though postmenopausal muscle loss is a phenomenon known to affect fast-twitch muscle fibers more than slow-twitch fibers, Urocortin seems to be a promising substance for muscle tissue in estrogen-deficient organism.

The diameter of oxidative muscle fibers increased after UCN treatment in *M. soleus* compared to the SHAM, OVX, and OVX+Estradiol groups. The ratio of capillaries to muscle fibers was higher in *M. soleus* after UCN high treatment, whereas parameters of muscle damage in serum were not altered due to urocortin-treatment. Bone parameters were not improved significantly, whereas parameters of metabolism during urocortin treatment, including increased cholesterol and triglycerides, should be examined more deeply in future studies. Urocortin is a promising substance for further studies regarding skeletal muscle, but its optimal dose of application and potential harmful effects on healthy and diseased tissues need to be further characterized.

## Limitations

In our model, functional muscle tests like strength test, wire hang test or different activity tests were not implemented. Therefore, the assertions regarding functional muscle parameters, although indirectly obtained by muscle enzyme activity and structural parameters, are missing.

There was no formal, blinded analysis of behavior: future studies should formally assess the effects of UCN treatment on home cage locomotor activity since effects on locomotor activity could affect muscle physiology.

Since this study does not include sarcopenic rats, the described effects just show the influence of UCN on skeletal muscle in an estrogen deficient osteoporotic rat model. Furthermore, the weight of *M. longissimus* was not measured which is also a limiting factor in this study. The effect of UCN on skeletal muscle in the OVX rat is limited compared to denervated or casted mice since the latter leads to a well-defined muscle atrophy, while the former mimics the estrogen-deficit in postmenopausal women.

Measured bone parameters are just static and do not imply analysis of osteoblast and osteoclast relation or bone turnover. Ultimate statements regarding the safety of UCN cannot be made until all possible side effects would be investigated in detail.

## Ethics Statement

The experimental design was approved by the local institutional animal care and use committee (district authorities of Oldenburg, Germany, registration numbers: 33.9-42502-04-10/0246).

## Author Contributions

DS, LG, TG, DH, MT, and MK conducted the experiments. DS, MT, SS, and MK interpreted the results. The final manuscript was drafted by DS, reviewed by SS and MK, and approved by all authors.

### Conflict of Interest Statement

The authors declare that the research was conducted in the absence of any commercial or financial relationships that could be construed as a potential conflict of interest.

## Abbreviations

ActRIIB-Fc: activin decoy receptor IIB
ALT: alanine transaminase
AST/GOT: aspartate aminotransferase/glutamic oxaloacetic transaminase
BMD: bone mineral density
BMP: bone morphogenetic protein
BV/TV: bone volume fraction
BW: body weight
CK: creatine kinase
CRF: corticotropin-releasing factor
CRF2R: corticotropin-releasing factor 2 receptor
Ct. Ar: cortical bone area
ERT: Post-menopausal estrogen replacement therapy
HDL: high-density lipoprotein
M.: musculus
MG: musculus gastrocnemius
ML: musculus longissimus
Mm.: musculi
MS: musculus soleus
N.Nd: trabecular nodes
NON-OVX: non-ovariectomy
OVX: ovariectomy
PAS: periodic acid–Schiff
s.c.: subcutaneous
SEM: standard error of the mean
Tb.N: trabecular number
Tb.N/Nd: mean trabecular junctions at one node
Tb.Th: trabecular thickness
UCN: urocortin-I.
