# Supplementary material for: Changes in Musculoskeletal System and Metabolism in Osteoporotic Rats Treated With Urocortin
Source: Front Endocrinol (Lausanne). 2019 Jun 24;10:400. doi: 10.3389/fendo.2019.00400 (PMC6601316; doi:10.3389/fendo.2019.00400)
Supplement: Supplementary file 1 [file Table_1.DOCX]

**Supplemental Table 1.** F statistics, degrees of freedom and p-values for one-way ANOVAs

| Parameters | F_df treatment,df residual_=F-value | p-value |
| --- | --- | --- |
| **Fig. 2** |  |  |
| Fat tissue | F_4,52_=10.00 | p<0.0001 |
| Heart | F_4,53_=4.699 | p=0.0025 |
| Liver | F_4,53_=22.34 | p<0.0001 |
| Kidney | F_4,52_=7.028 | p=0.0001 |
| Spleen | F_4,52_=11.50 | p<0.0001 |
| M. gastrocnemius, weight | F_4,52_=4.727 | p=0.0025 |
| M. gastrocnemius, weight/bw | F_4,52_=7.161 | p=0.0001 |
| M. soleus, weight | F_4,51_=11.73 | p<0.0001 |
| M. soleus, weight/bw | F_4,51_=6.299 | p<0.0003 |
|  |  |  |
| **Fig. 3** |  |  |
| M. gastrocnemius, diameter I | F_4,37_=1.457 | p=0.2349 |
| M. gastrocnemius, diameter II | F_4,37_=0.7419 | p=0.5696 |
| M. longissimus, diameter I | F_4,38_=2.534 | p=0.0441 |
| M. longissimus, diameter II | F_4,38_=2.476 | p=0.0605 |
| M. soleus, diameter I | F_4,50_=7.355 | p<0.0001 |
| M. gastrocnemius, cap/fiber | F_4,31_=2.897 | p=0.0380 |
| M. longissimus, cap/fiber | F_4,41_=2.195 | p=0.5688 |
| M. soleus, cap/fiber | F_4,43_=2.432 | p=0.0619 |
|  |  |  |
| **Fig. 4** |  |  |
| LDH, M. gastrocnemius | F_4,35_=1.236 | p=0.3135 |
| LDH, M. longissimus | F_4,35_=0.3476 | p=0.8439 |
| LDH, M. soleus | F_4,35_=0.5019 | p=0.7345 |
| CS, M. gastrocnemius | F_4,35_=1.962 | p=0.1219 |
| CS, M. longissimus | F_4,35_=0.2921 | p=0.8811 |
| CS, M. soleus | F_4,35_=3.139 | p=0.0263 |
| CI, M. gastrocnemius | F_4,34_=3.064 | p=0.0294 |
| CI, M. longissimus | F_4,35_=1.290 | p=0.2928 |
| CI, M. soleus | F_4,34_=2.403 | p=0.0689 |
|  |  |  |
| **Fig. 5** |  |  |
| CK | F_4,30_=1.375 | p=0.2661 |
| AST/GOT | F_4,29_=2.126 | p=0.1031 |
| ALT | F_4,30_=8.258 | p=0.0001 |
| Cholesterol | F_4,30_=7.441 | p=0.0003 |
| Glucose | F_4,30_=36.70 | p<0.0001 |
| Uric acid | F_4,25_=4.248 | p=0.0093 |
| HDL | F_4,30_=9.029 | p<0.0001 |
| Triglyceride | F_4,30_=4.339 | p=0.0069 |
|  |  |  |
| **Table 1** |  |  |
| **Biomechanics** |  |  |
| Stiffness | F_4,53_=1.389 | p=0.2504 |
| Yield Load | F_4,53_=7.020 | p=0.0001 |
| Maximum Load | F_4,53_=7.138 | p=0.0001 |
| **Ashing** |  |  |
| Ca^2+^ | F_4,50_=3.646 | p=0.0111 |
| PO_4_^3-^ | F_4,50_=1.674 | p=0.1708 |
| Ca^2+^/PO_4_^3-^ | F_4,50_=1.862 | p=0.1318 |
| Inorganic mass | F_4,50_=11.34 | p<0.0001 |
|  |  |  |
| Parameters | F_df treatment,df residual_=F-value | p-value |
| **Micro-CT** |  |  |
| Total BMD | F_4,53_=22.94 | p<0.0001 |
| Bone volume | F_4,53_=41.07 | p<0.0001 |
| Total volume | F_4,53_=2.145 | p=0.0881 |
| BV/TV | F_4,53_=45.13 | p<0.0001 |
| Tr.+Ct. BMD | F_4,53_=6.488 | p=0.0003 |
| Tr. BMD | F_4,53_=6.216 | p=0.0004 |
| Ct. BMD | F_4,53_=5.645 | p=0.0007 |
| Tb.N. | F_4,53_=15.69 | p<0.0001 |
| N.Nd. | F_4,53_=17.53 | p<0.0001 |
| Tb.N/Nd | F_4,53_=10.77 | p<0.0001 |
| Tb.Sp. | F_4,53_=2.465 | p=0.0561 |
| Tb.Th. | F_4,53_=19.43 | p<0.0001 |
| Ct.Ar. | F_4,53_=1.044 | p=0.3934 |
| E.Ar. | F_4,53_=1.086 | p=0.3728 |
| T.Ar. | F_4,53_=1.120 | p=0.3572 |
